# Symptoms and quality of life among men starting treatment for metastatic castration-resistant prostate cancer – a prospective multicenter study

**DOI:** 10.1186/s12904-024-01410-w

**Published:** 2024-03-27

**Authors:** Ulrika Rönningås, Maja Holm, Per Fransson, Lars Beckman, Agneta Wennman-Larsen

**Affiliations:** 1 Department of Nursing, Umeå University, Umeå, 901 87 Sweden; 2 Department of Oncology, Sundsvall County Hospital, Sundsvall, Sweden; 3 Department of Nursing Sciences, Sophiahemmet University, Stockholm, Sweden; 4 Department of Health Care Sciences, Palliative Research Centre, Marie Cederschiöld University College, Stockholm, Sweden; 5 Department of Radiation Sciences, Umeå University, Umeå, Sweden; 6 Division of Insurance Medicine, Department of Clinical Neuroscience, Karolinska Institutet, Stockholm, Sweden

**Keywords:** Prostatic neoplasm, Metastatic castration-resistant prostate cancer, Symptom management, Symptom burden, MSAS, Quality of life, Early integrated palliative care

## Abstract

**Background:**

Men with metastatic castration-resistant prostate cancer (mCRPC) have an incurable disease. Along with prolonging life, symptom management is one of the main goals with treatment. This is also important from a palliative care perspective where the life prolonging outcomes should be balanced with quality of life (QoL) in this late phase. It is also essential in symptom management to view different dimensions of symptoms, for example how severe or distressing symptoms are, to support best QoL. Therefore, more knowledge is needed about the symptom experience when these treatments are initiated and thus the aim of this study was to describe different dimensions of symptoms in men with mCRPC starting their first-line of life-prolonging treatment, and to describe the association between symptom burden and QoL.

**Methods:**

Baseline data from a prospective longitudinal study of 143 men with mCRPC starting their first-line life-prolonging treatment were used. Symptoms were measured using the Memorial Symptom Assessment Scale (MSAS) and global QoL was measured by the EORTC QLQ C-30. Data was analyzed using descriptive- and multivariable linear regression analyses.

**Results:**

On average, the men had more than 10 symptoms (range 0–31 of 33). 50% or more reported sweats, lack of energy, pain, problems with sexual activity and sexual desire. The symptoms they reported as most severe, or most distressing were not always the ones that were reported as most frequent. There was an association between QoL and physical symptoms, and also between QoL, and analgesic use and prostate-specific antigen (PSA) values.

**Conclusion:**

Even if some men with mCRPC report many symptoms, the dimensions of severity and distress levels vary, and the most frequent symptoms was not always the most burdensome or distressing. There was an association between high physical symptom burden and QoL, suggesting that it is not the number of symptoms that affects QoL but rather the subjective perceived impact of the physical symptoms experienced. The knowledge of how men with mCRPC experience and perceive their symptoms may help health care professionals in symptom management aiming to improve QoL, which is a cornerstone in integrating early palliative care.

## Introduction

Prostate cancer had the third highest incidence of all cancers in 2020 with 1.4 million new cases [1]. In Sweden the overall survival for men with metastatic castration-resistant prostate cancer (mCRPC) is 13.2–23.2 months depending on whether having metastases already at diagnosis or not [2]. The overall survival has improved over the last decade due to several new treatment options [3–8] that may prolong life, manage symptoms, and improve quality of life (QoL). Symptom management and improvement of QoL are also fundamental aspects of a palliative care approach that should be integrated early along with life-prolonging treatments in life-limiting disease [9, 10].

Even though many patients have a wish to prolong life [11], men with mCRPC express that it is also important to weigh this against QoL [12]. Patients with advanced cancer often suffer from unmet both psychological and physical needs [13]. Unmet needs regarding symptoms and QoL have been found among men with metastatic prostate cancer in a Swedish context [14]. A need to integrate psychosocial support as a part of routine care has also been expressed by men with advanced prostate cancer, meaning that they do not have to advocate these needs by themselves [15].

Since a substantial symptom burden can be experienced when prostate cancer progresses to a mCRPC phase, it is important to have knowledge about the men’s symptoms when starting life-prolonging treatment. The information about that the disease has progressed may be overwhelming for the men and fear/uncertainty about the future has also been shown [15]. The perception of symptoms is often multidimensional [16] and symptom burden has been defined as “the subjective, quantifiable prevalence, frequency, and severity of symptoms placing a physiologic burden on patients and producing multiple negative, physical and emotional patient responses” [17]. A multidimensional assessment of frequency, severity and distress provide more information than if only one dimension is assessed. The three dimensions may be measured separately or together. Less frequent symptoms can be experienced as very severe and/or distressing and vice versa [18]. Thus, it is important to consider the multidimensional perspectives of symptom burden with a focus on frequency, severity and distress [19].

In qualitative studies [20–22], men with mCRPC have described numerous symptoms, of which pain and fatigue were the worst. Pain originating from bone metastases, – the predominant site for distant metastases – can be severe and affect daily activities as well as sleep and mood [23]. In a large international study of 927 men with advanced prostate cancer, bone pain, fatigue, urinary problems, and sexual dysfunction were the most reported symptoms irrespective of having treatment or not [24]. In advanced disease it has also been reported that patients who experience certain symptoms e.g. pain in a specific location or blood in stool/urine, sometimes attribute them to potential metastases [20]. Men closer to death also report more symptoms [14].

Physical and psychological symptoms can affect QoL. In one study almost 75% of the men with mCRPC reported fatigue, about half of them reported moderate to severe fatigue, which was associated with lower QoL [25]. Low QoL has also been reported for men with metastatic prostate cancer six months before death [14]. Burbridge et al. [20] showed that one of the areas most impacted by a metastatic prostate cancer was emotional well-being. The men in the study mentioned feelings of worry, anxiety, depression, fear, frustration, and anger, feelings they related to the metastatic disease [20]. Worry and anxiety have also been shown before receiving PSA values [26] and an association between distress and an increasing prostate-specific antigen (PSA) value has been found [27]. Some men wanted to understand how the disease would progress, what impact it would have on their QoL, and how much time they had left [15].

Although survival for men with mCRPC has improved with the rapid increase in treatment options over the last 15 years, the disease is still life-limiting and a palliative care approach with active symptom management aiming to improve QoL is important. Most previous studies that describe symptoms when starting life-prolonging treatments are clinical trials with narrow inclusion criteria. Knowledge about multidimensional symptom burden in a real world-situation is important as a basis for appropriate symptom management. To our knowledge, there is only one study [28] describing the symptom burden of men with mCRPC starting life-prolonging treatment in a real-world situation, and no study that has used an instrument that assesses more than one or two dimensions. Therefore, the aim of the present study was to describe different dimensions of symptoms in men with mCRPC starting their first-line of life-prolonging treatment, and to describe the association between symptom burden and QoL in this group of men.

## Methods

### Study design

This cross-sectional study was based on baseline data from a longitudinal, prospective multicenter project [12, 29] of 154 men with mCRPC starting life-prolonging treatment regarding their experiences, expectations, and decision making in relation to treatment. Inclusion criteria were men who were about to start their first-line treatment for mCRPC, and who could understand and express themselves in Swedish. A power analysis based on clinically relevant changes in one of the instruments (not used in this study) [30] was conducted for the overall project. A sample of 120–150 men was shown to be sufficient. For the analysis in the present study, a sample size of above 100 were considered sufficient in detecting associations of medium effect [31].

The men were included between April 2015 and March 2022, from four oncology departments in Sweden, located at both university hospitals and county hospitals. In conjunction with treatment start, eligible participants received written and oral information by the treating physician and/or a research nurse. If accepting participation, he signed an informed consent and was then given the baseline questionnaire together with a pre-paid envelope.

### Data collection

In the present study, questionnaire- and medical data from the baseline questionnaire that were returned by 143 out of the 154 men, were used.

### Measures

The questionnaire includes demographic questions and well-validated instruments regarding symptoms, and QoL.

Symptom burden were measured using the Memorial Symptom Assessment Scale (MSAS) [18]. The MSAS was developed to provide multidimensional information about 32 physical and psychological symptoms experienced in the last seven days. For 24 of these symptoms three dimensions (frequency, severity, distress) are measured. For the other eight symptoms two dimensions are measured (severity and distress). Frequency and severity are measured on a four-point rating scale while distress is measured on a five-point rating scale which is converted to a four-point scale prior to analysis [18]. Higher scores indicate greater frequency, severity, and distress. An overall symptom score for each symptom, including frequency, severity, and distress, are then calculated [18]. Based on some of the symptom scores three subscales (PHYS, PSYCH, GDI) are calculated with scores 0–4. The MSAS-PHYS subscale contains 12 physical symptoms (lack of appetite, lack of energy, pain, feeling drowsy, constipation, dry mouth, nausea, vomiting, change in taste, weight loss, feeling bloated, dizziness). The MSAS-PSYCH subscale contains six symptoms (feeling sad, worrying, feeling irritable, feeling nervous, difficulty sleeping, difficulty concentrating). The third subscale is a global distress index, MSAS-GDI, not used in the present study.

For this study the question “problems with sexual activity and desire” was split into two questions since all these men are medically or surgically castrated and the activity or desire may differ from in a non-castrated population, thus here the MSAS consists of 33 symptoms. As a measure of symptom burden the number of symptoms experienced (0–33 symptoms) and the two subscales MSAS-PHYS and MSAS-PSYCH were used. Even if only 18 of the 33 symptoms from MSAS are used in the two subscales used here, all symptoms were included in the count of the number of symptoms. Cronbach’s alpha, for MSAS-PHYS was 0.814 and for MSAS-PSYCH 0.803.

Self-reported QoL was measured using the global QoL subscale from the EORTC QLQ C-30 questionnaire [32, 33]. The subscale is based on two questions: ”How would you rate your overall health during the past week?” and “How would you rate your overall quality of life during the past week?” with response alternatives on a likert scale, with anchor points 1 = “poor” and 7 = “excellent”. The responses were transformed to a 0-100 scale according to the scoring manual [34], where higher points indicate higher global QoL. Cronbach’s alpha was 0.940.

Covariates included in the multivariable analysis were self-reported age, educational level (categorized as elementary school, high school, university), and from the medical records the latest taken PSA-value, time since diagnosis of metastatic disease, and use of analgesics (yes/no).

### Data analysis

Missing values were managed according to the scoring guidelines for the respective questionnaires [18, 34]. Comparisons between medical data for those included, i.e., those who answered the first questionnaire, and those not included (who did not return the baseline questionnaire) were conducted using Mann-Whitney U-test and Chi2-test for continuous and categorical data, respectively. Frequencies and proportions are presented for demographic and medical characteristics. First a bivariate linear regression was applied to assess the associations between symptom burden (number of symptoms, MSAS-PHYS and MSAS-PSYCH subscales) and QoL but also between the covariates and QoL (Model 1). Then, the variables showing a significant (*p* < 0.05) association in the bivariate analysis (Model 1) were included simultaneously in a multivariable regression analysis (Model 2). The assumptions for linear regression were evaluated using the normal P-P plots, scatterplots of residuals and evaluation of variance inflation factor (VIF) statistics, and all assumptions were met according to these measures [31]. Since more than half of the men had started treatment when answering the questionnaire, a subgroup analysis using Mann Whitney U-test was performed to investigate if there were differences in symptom burden reported between those who had and those who had not started treatment. For all analyses, *p* < 0.05 was considered statistically significant. Data analysis was conducted using IBM SPSS 27 (Armonk, NY: IBM Corp).

## Results

### Sample characteristics

**Fig. 1:**
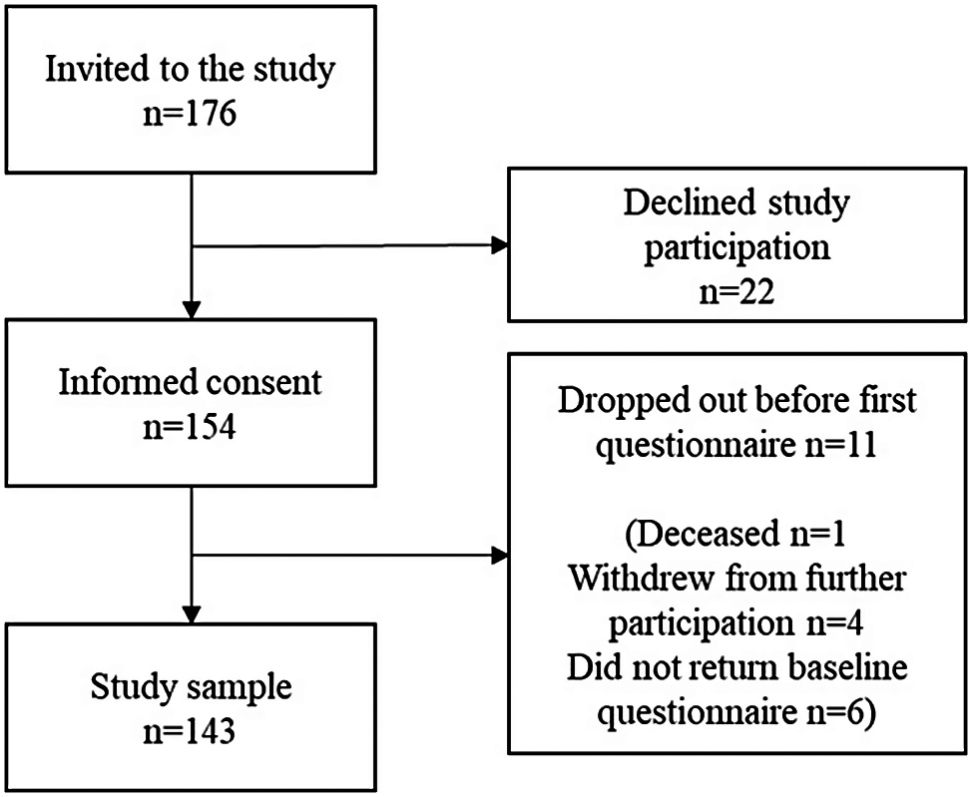
Study enrollment flow-chart

Of the 154 men who accepted to participate in the study, 11 did not return the baseline questionnaire and were thus not included in the analyses (Fig. 1). There were no significant differences between those who returned the questionnaire and those who did not regarding; age (*U* = 716, *p* = 0.620), PSA-values (*U* = 982, *p* = 0.170), time since primary diagnosis and time since metastatic disease (*U* = 798, *p* = 0.842, *U* = 698, *p* = 0.631), analgesic use (χ2 = 0.697, *p* = 0.325) or tumor status (T, N, M, and Gleason score) (ranging between χ2 = 0.319˗5.062, *p* = 0.653˗0.989).

The mean age of the included 143 men was 75 years (SD 7.2), ranging between 50 and 88 years at the time of inclusion (Table 1).

**Table 1:** Sociodemographic and medical characteristics of 143 men with metastatic castration-resistant prostate cancer: frequencies, percentages, mean, standard deviation (SD), min-max

**Age (years)**			Mean (SD)	75.0 (7.2)		
			Min-max	50–88		
**Years since primary diagnosis**			Mean (*SD*)	4.6 *(4.7)*		
			Min-max	0–22		
			Missing	0		
**Years since diagnosis of metastatic disease**			Mean (*SD*)	1.3 *(1.9)*		
			Min-max	0-13.3		
			Missing	*n* = 1		
**PSA (ng/ml)**			Mean *(SD)*	87.8 *(219)*		
			Min-max	0.50–2082		
			Missing	0		
				**n**	**%**	
**Marital status**			Married/had a partner	112	78.3	
			Single/widowed,	29	20.3	
			Missing	2	1.4	
**Education**			Elementary school	63	44.1	
			High school	38	26.6	
			University	41	28.7	
			Missing	1	0.7	
**Tumor (T) stage**			T1	12	8.4	
			T2	29	20.3	
			T3	70	49.0	
			T4	21	14.7	
			Tx	6	4.2	
			Missing	5	3.5	
**Node (N) stage**			N0	81	56.6	
			N1	44	30.8	
			Nx	13	9.1	
			Missing	5	3.5	
**Metastasis (M) stage**			M0	76	53.1	
			M1	63	44.1	
			Mx	1	0.7	
			Missing	3	2.1	
**Gleason Score**			6	13	9.1	
			7	46	32.2	
			8	35	24.5	
			9	34	23.8	
			10	2	1.4	
			Missing^§^	13	9.1	
**Metastasis site**			Bone	96	67.1	
			Lymph nodes	40	28.0	
			Lung	2	1.4	
			Liver	2	1.4	
			Other	1	0.7	
			Missing	2	1.4	
**Treatment**			Abiraterone	22	15.4	
			Docetaxel	40	28.0	
			Enzalutamide	74	51.7	
			Radium-223	4	2.8	
			Cabazitaxel	3	2.1	
**Analgesic use**			Yes/no	69/74	48.3/51.7	

^(§)^ No biopsy

Most men were married/had a partner (78%, *n* = 112), and 55% (*n* = 79) had a high school or university education. The PSA-values ranged from 0.5 to 2082 ng/ml. The mean time since the men were first diagnosed with prostate cancer were 4.6 years (SD 4.7) and 44.1% (*n* = 63) of the men already had metastatic disease upon diagnosis. All men were about to start (35%) or had recently started (65%) a life-prolonging treatment for mCRPC. In most cases the treatment was a second-generation antiandrogen (enzalutamide 51.7%, *n* = 74 or abiraterone 15.4%, *n* = 22) or chemotherapy (docetaxel 28.0%, *n* = 40 or cabazitaxel 2.1%, *n* = 3), and in a few cases (2,8%, *n* = 4) radium-223. Of all men, 48.3% (*n* = 69) were currently being treated with analgesics, and of those reporting pain 63.9% (*n* = 46) were using analgesics. There were no significant differences between those using and not using analgesics in any of the pain dimensions (frequency: χ2 = 0.883, *p* = 0.347, severity: χ2 = 0.531, *p* = 0.466, distress: χ2 = 0.610, *p* = 0.435).

### Description of symptom burden and QoL

On average the men reported 10.6 symptoms (SD 7.2) (Table 2).

**Table 2:** Symptom burden, and QoL of 143 men with metastatic castration-resistant prostate cancer: mean, standard deviation (SD), min-max

	Mean (SD)	Min-max
**Number of symptoms**	10.6 *(7.2)*	0–31
**Physical symptoms (MSAS-PHYS subscale)**	0.50 *(0.50)*	0-2.06
**Psychological symptoms (MSAS-PSYCH subscale)**	0.48 *(0.61)*	0-3.20
**Global QoL**	63.6 *(22.2)*	0-100

**Fig. 2:**
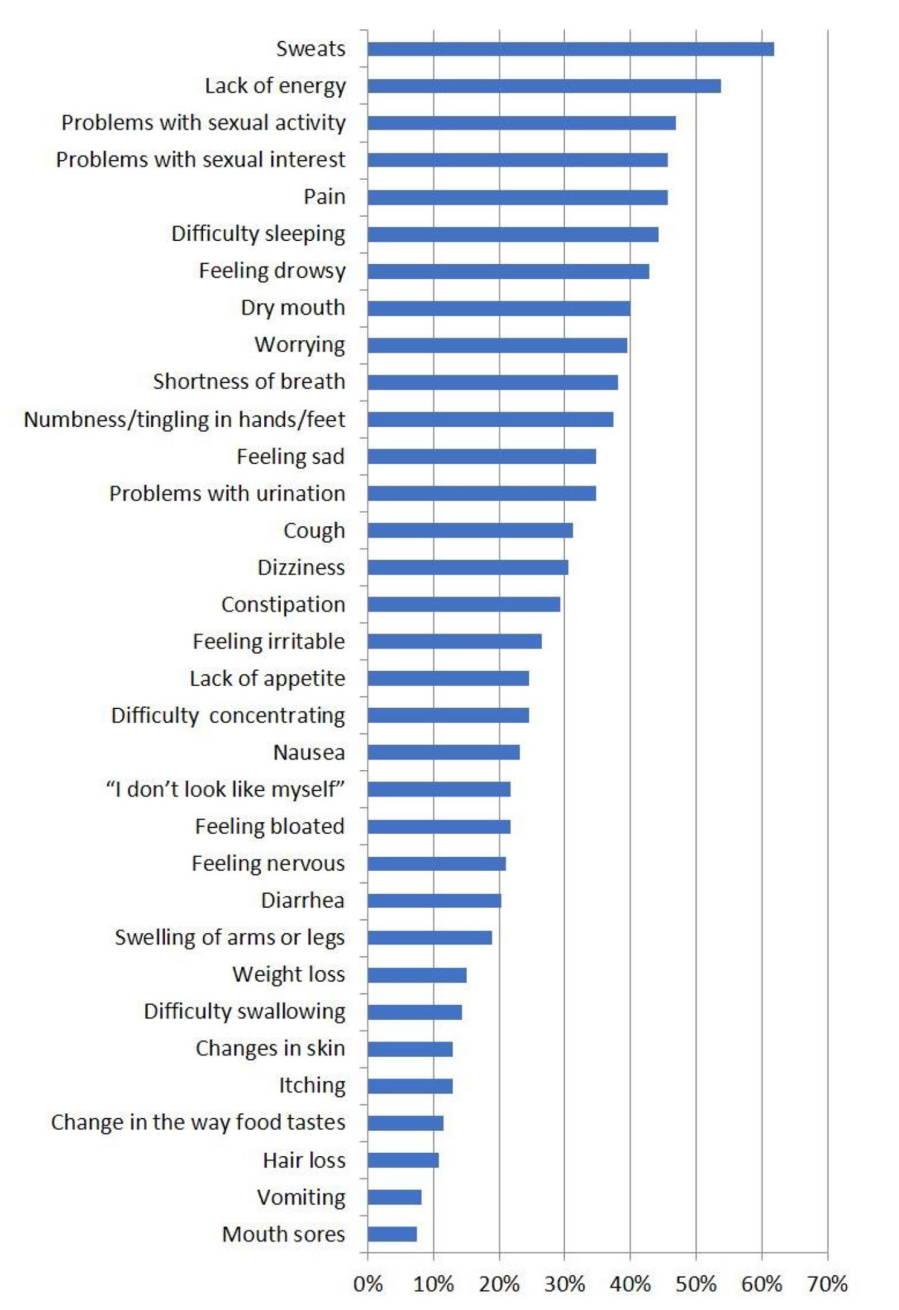
Percentages of men (*n* = 143) reporting having experienced the 33 symptoms listed in MSAS. More than one symptom can be reported

Some men had no symptoms at all (*n* = 3, 2.1%) while 37% (*n* = 51) men had between 10 and 21 symptoms and one man reported 31 symptoms. The most frequently reported symptoms were physical. 50% or more reported sweats, lack of energy, problems with sexual activity and sexual desire and pain, and more than 40% reported difficulty sleeping, feeling drowsy, dry mouth, worrying, shortness of breath and numbness/tingling in hands and feet (Fig. 2).

**Fig. 3:**
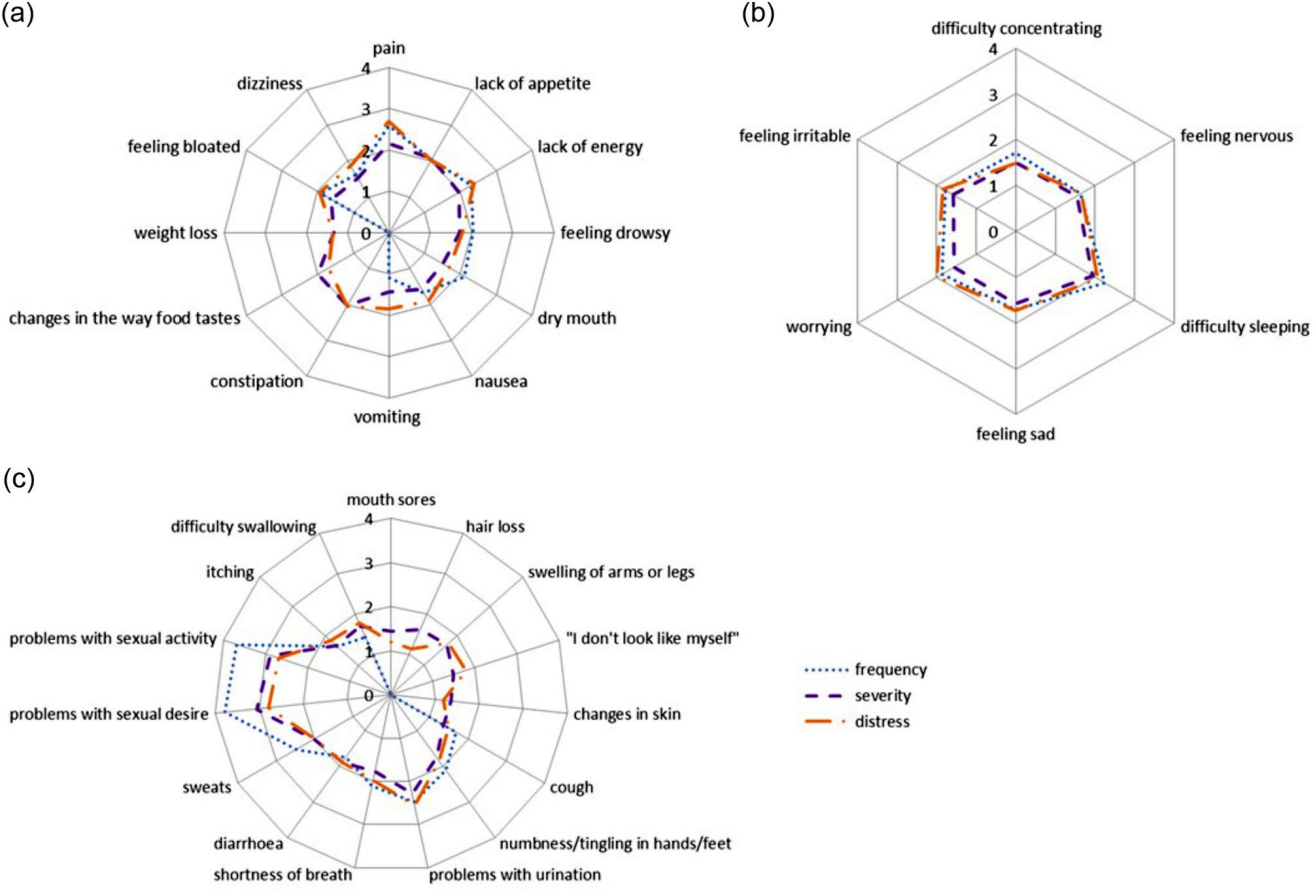
(**a**) MSAS-PHYS subscale dimension scores, (**b**) MSAS-PSYCH subscale dimension scores (**c**) dimension scores for symptoms from the MSAS not present in PHYS or PSYCH subscales

Less than 15% reported hair loss, vomiting and mouth sores. When it comes to multidimensional aspects of symptoms, the levels of frequency, severity and distress varies. For pain, the levels of distress were slightly higher than the levels of frequency, which in turn was higher than the levels of severity (Fig. 3a). Regarding vomiting the frequency was low while the severity was higher, and the levels of distress even higher. Nausea also had higher distress scores, although the prevalence was low (Fig. 3a). Sexual problems were often reported and there was a large difference between frequency and severity and distress, where the level of distress was the lowest (Fig. 3c). The psychological symptoms were overall not reported as so distressing or severe (Fig. 3b) even if difficulty sleeping and worrying were among the top nine most frequently reported symptoms. All MSAS symptom dimensions are shown in (Fig. 3a-c).

For physical symptoms (the MSAS-PHYS subscale) the mean score was 0.50 (SD 0.50) and for psychological symptoms (the MSAS-PSYCH subscale) 0.48 (SD 0.61) (Table 2). The mean global QoL score was 63.6 (SD 22.2) and 10% (*n* = 14) reported the highest level (100). 19% (*n* = 27) reported 50 and below and of these some men (*n* = 8, 5.6%) experienced very low levels of QoL with scores ranging between 0 and 25. There were no significant differences between those who had started treatment when answering the questionnaire and those who had not regarding number of symptoms, MSAS-PHYS and MSAS-PSYCH (*U* ranging between 2006 and 2261, *p* = 0.228–0.952).

### Associations between symptom burden and QoL

In bivariate analyses (Model 1) there were significant associations between low QoL and high number of symptoms (β= -0.389, p  < 0.001), as well as high physical (β= -0.617, p<0.001) and psychological symptom burden (β= -0.341, p  < 0.001). Higher PSA values (β= -0.209, *p* = 0.012) and the use of analgesics (β= -0.223, *p* = 0.007) were also associated to low QoL (Table 3). The overall multivariable linear regression model (Model 2) was statistically significant (R^2^_adj_ = 0.407, *p* < 0.001) and with a VIF of 1.018–1.861. An association was found between having high physical symptom burden (β= -0.544, p  < 0.001) and global QoL. There were also an association between both use of analgesics (β= -0.153, *p* = 0.021) and global QoL, higher PSA values (β= -0.143, *p* = 0.033) and global QoL. No significant associations were found between number of symptoms (β= -0.055, *p* = 0.503) or psychological symptoms (β = 0.019, *p* = 0.820) and QoL.

**Table 3:** Bivariate and adjusted regression coefficients with 95% confidence interval (95%CI) for associations between QoL and symptom burden adjusted for sociodemographic factors, medical factors, and analgesic use

	Model 1^#^				Model 2^$^			
Variable	B		β	p	B	95% CI	β	p
Global QoL [constant]					82.509	[76.76, 88.26]		
Number of symptoms	-1.201		− 0.389	< 0.001	− 0.171	[-0.675, 0.333]	− 0.055	0.503
Physical symptoms	-27.634		− 0.617	< 0.001	-24.363	[-32.172, -16.555]	− 0.544	< 0.001
Psychological symptoms	-12.467		− 0.341	< 0.001	− 0.688	[-6.656, 5.279]	0.019	0.820
Age (years)	0.200		0.065	0.443				
Highest educational level (elementary school/high school)	− 0.606		− 0.012	0.895				
Highest educational level (elementary school/ university)	-4.917		− 0.100	0.275				
Time since metastatic disease (in months)	0.040		0.042	0.622				
PSA (ng/ml)	− 0.021		− 0.209	0.012	− 0.015	[-0.028, − 0.001	− 0.143	0.033
Analgesic use	-9.902		− 0.223	0.007	-6.767	[-12.486, -1.048]	− 0.153	0.021

*Note* For model 2: R^2^_adj_ = 0.407 (*N* = 143, *p* = 0.001). CI = Confidence Interval for B

^(#)^ Model 1: Bivariate association between all variables and global QoL

^($)^ Model 2: Association between global QoL and all significant independent variables in Model 1

## Discussion

The aim of this study was to describe different dimensions of symptoms in men with mCRPC, starting their first-line of life-prolonging treatment, and to describe the association between symptom burden and QoL. The results show that many symptoms were reported when starting life-prolonging treatments, although it was not the most often reported symptoms that the men perceived as most severe or distressing. An association between the number of symptoms, physical symptoms, psychological symptoms and QoL was found. In the multivariable analysis and when adjusted for sociodemographic and medical factors, physical symptoms were independently associated with QoL as were PSA values and analgesic use. But interestingly, neither the number of symptoms nor psychological symptoms remained significantly associated with QoL.

The men in the present study reported a rather large variation in number of symptoms, from no symptoms at all to up to 31, with a mean of 10.6 symptoms. This corresponds with the average number of symptoms reported by patients in different stages of colorectal cancer receiving chemotherapy (mean 10.3) [35], but are more symptoms than men and women over 75 years with multimorbidity report (mean 8.5) [36]. It is also relevant to compare the results with men with newly diagnosed prostate cancer that report in average 5.5 symptoms [37]. The relatively high number of reported symptoms in the present study may be explained by the fact that these men were in a progressive phase of the disease. The progression may have been causing new symptoms, which may not yet have been given attention, or of which the treatment is part of the symptom management. However, it may also be a sign of inadequate symptom management. Many of these men have been living long with their disease and may not have been followed up systematically with symptom assessments. Even though, in Sweden, all men diagnosed with prostate cancer should be assigned a contact nurse, the contact is need based. In case of new symptoms or problems, the initiative to contact relies on the patient [38]. When using a structured assessment, other symptoms may be found that are not reported in a clinical situation when asking in a more open way about symptoms.

The multidimensional aspects of frequency, severity and distress varied between the different symptoms. In our study the distress dimension score for pain was higher than the frequency and severity dimension scores. It was surprising that 50% of the men experienced pain, and although 48% of the men were under analgesic treatment the pain management was not sufficient. In another recent study of men with mCRPC in a real-world situation, 55% reported pain even if the severity of their reported pain was low [28]. All men in this study had metastases and a majority had bone metastases which together with pain may cause lack of energy and difficulty sleeping [23]. These three symptoms were also among the top six reported. A recently published study about patients with different types of advanced cancer [39] also showed that these three symptoms were among the most common regardless of whether the patients were classed as having low, moderate or high symptom burden.

It was not always the most frequent or severe symptom that was the most distressing. Vomiting was one of the least reported symptoms but the levels of distress for most of those experiencing vomiting was high. This show that the assessment of different symptom dimensions could be used in clinical situations as a basis for improved symptom management to help identifying the symptoms that are the most burdensome [40]. In this group of men living their last years of life assessment of different symptom dimensions to enhance effective symptom management may be an important aspect towards integrating early palliative care with oncological treatment [9].

Neither the number of symptoms or psychological symptoms were independently associated with QoL, while high physical symptom burden were associated to low QoL together with higher PSA values and the use of analgesics. These three factors may all indicate a more advanced disease. Previous studies [22, 41] have shown that symptom burden, both physical and psychological, may increase and QoL decrease when men with prostate cancer move to a mCRPC phase. Symptoms such as pain have been described as triggering thoughts and fears about the consequences of a potential disease progression, such as being dependent on others and what dying would be like [29]. In the lack of symptoms, men with prostate cancer describe PSA-values as the only indicator they have of eventual disease progression [29, 42, 43]. PSA-values may therefore provoke worry and anxiety [26, 44]. In this progressive late phase of the disease, it has been shown that psychological symptoms are associated to QoL [15, 45]. For that reason, we also expected that psychological symptoms should have been associated to QoL. On the contrary, we found no such association. One explanation for this may be that the start of life-prolonging treatment can give hope and even if the men experience certain psychological symptoms, this may reduce the effect of the psychological symptoms on QoL. In a study of women with breast cancer undergoing late lines of chemotherapy they expressed that their hope grew stronger during treatment [46].

Many of the men reported a high symptom burden, both in terms of number of symptoms and in levels of frequency, severity and distress, and from a palliative care perspective, symptom management and QoL are important [10, 47]. A palliative care approach, with active symptom management should be implemented early in the disease trajectory in advanced cancer [9]. It has also been shown that when a combination of both palliative and oncologic approaches is utilized, both QoL and symptom control are improved [48]. Furthermore, patients perceived a more satisfactory healthcare experience when palliative care was provided in conjunction with oncological treatments [49].

### Study limitations

Even if the sample was relatively small, a strength in this study is the multicenter real-world recruitment of patients and that only few men declined participation. Most other studies of this group are clinical trials which use strong selection criteria [3–8] for example regarding fitness for all types of treatment including chemotherapy. This rendered a somewhat older sample than those normally recruited for studies of mCRPC which may be more representative of the group since men not fit for chemotherapy were able to participate. Thus, the most common treatment was enzalutamide. In a study of treatment utilization in a Swedish context between the years 2006 and 2016 the first choice of treatment was docetaxel followed by enzalutamide [50], but second-generation anti-androgens have been more commonly used in recent years, thus giving men unfit for chemotherapy a treatment option.

A strength, of this study is the multidimensional approach of the MSAS questionnaire, which gave a thorough symptom burden assessment. However, the severity and distress dimensions had somewhat higher missing rates (data not shown), which may indicate that the multidimensionality of the MSAS may have been misunderstood by the men. A mixed methods approach using interviews could also have given a deeper understanding of the different dimensions from the men’s perspective and could be recommended in future studies. Although missing values were managed according to the questionnaire guidelines [18] they may have affected the results in some systematic way.

Another limitation may be that half of the men already had started treatment when answering the questionnaire due to that they were late in returning the questionnaire. However, no significant differences were found regarding number of symptoms, MSAS-PHYS and MSAS-PSYCH between those who had started treatment when answering the questionnaire and those who had not. An explanation may be that since most men had a second-generation antiandrogen treatment (67%) most symptoms from the treatment may arise later in the treatment trajectory.

A strength is that medical data were collected from the participants’ medical records, including retrospective data from the time of the prostate cancer diagnosis. This gave a thorough view of the sample and a possibility to analyze if there were differences regarding medical factors between the men who returned the baseline questionnaire and those who did not.

## Conclusion

Even if some men with mCRPC report many symptoms, the dimensions of severity and distress levels vary, and the most frequent symptoms may not be most burdensome or distressing. Only high physical symptom burden was associated to QoL, while psychological were not. This suggests that it is not the number of symptoms that affects QoL but rather the subjective impact of the physical symptoms experienced. The knowledge on how the men with mCRPC experience and perceive their symptoms, will help health care professionals in symptom management aiming to improve QoL, which is a step in integrating early palliative care. Future studies of this understudied group may benefit from a longitudinal approach to investigate changes over time regarding symptom burden and QoL when the prostate cancer progresses further.

## Data Availability

The datasets generated and/or analysed during the current study are not publicly available due to Swedish law and data regulations but are available from Agneta Wennman-Larsen on reasonable request.

## Abbreviations

mCRPC: Metastatic castration-resistant prostate cancer
MSAS: Memorial Symptom Assessment Scale
PSA: Prostate-specific antigen
QoL: Quality of life
